# Neuroprotective Potential of Aminonaphthoquinone Derivatives Against Amyloid Beta-Induced Neuronal Cell Death Through Modulation of SIRT1 and BACE1

**DOI:** 10.1007/s11064-024-04281-y

**Published:** 2024-12-07

**Authors:** Setthawut Apiraksattayakul, Ratchanok Pingaew, Veda Prachayasittikul, Waralee Ruankham, Tanawut Tantimongcolwat, Virapong Prachayasittikul, Supaluk Prachayasittikul, Kamonrat Phopin

**Affiliations:** 1 Center for Research Innovation and Biomedical Informatics, Faculty of Medical Technology, Mahidol University, Bangkok, 10700 Thailand; 2 Department of Chemistry, Faculty of Science, Srinakharinwirot University, Bangkok, 10110 Thailand; 3 Department of Clinical Microbiology and Applied Technology, Faculty of Medical Technology, Mahidol University, Bangkok, 10700 Thailand

**Keywords:** Alzheimer’s disease, Aminonaphthoquinone, SIRT1, Neuroprotection, Antioxidant

## Abstract

Alzheimer’s disease (AD) is characterized by the accumulation of tau protein tangles and amyloid-β (Aβ) plaques in the central nervous system (CNS), leading to progressive neurodegeneration. Hence, the discovery of disease-modifying agents capable of delaying the progression is essential for effective management. Aminonaphthoquinone (ANQ) is an attractive pharmacophore with various biological effects. This study explores the neuroprotective potentials of ANQ derivatives (**1**–**18**) using in vitro models of AD pathology (i.e., Aβ_42_-induced SH-SY5Y cells). Findings demonstrated that all compounds mitigated Aβ_42_-induced cellular damage by preserving cell viability and morphology. Among all, four compounds (**10**, **12**, **16**, and **18**) showed potent antioxidant activities as well as abilities to minimize AD-related damages (i.e. decreasing intracellular reactive oxygen species (ROS) production, preserving mitochondrial membrane potential (MMP), protecting membrane damage, and modulating beta-secretase 1 (BACE1) activity) with comparable protective effects to the well-known neuroprotectant, resveratrol (RSV). A molecular docking study indicated these compounds could suitably bind to sirtuin 1 (SIRT1) protein with preferable affinity. Key amino acid residues and key functional groups essential for binding interactions were revealed. Target prediction identified a list of possible AD-related targets of these compounds offering insights into their mechanisms of action and suggesting their multifunctional potentials. Additionally, in silico predictions revealed that these candidates showed favorable drug-like properties. Overall, this study highlighted the therapeutic potential of ANQ derivatives in AD treatment, emphasizing the need for further experimental validation and comprehensive investigations to fully realize their therapeutic benefits.

## Introduction

AD, a neurodegenerative phenomenon afflicting millions globally, represents a leading cause of dementia. AD is characterized by progressive deterioration in memory, cognition, and behavior as well as the accumulation of Aβ plaques in the brain [1]. While the precise origins of this devastating disease remain a mystery, the pivotal role of Aβ-induced oxidative stress has shed light on its pathogenesis and progression. The buildup of excessive Aβ accumulated in the brain is likely initiated by an increased enzymatic activity of BACE1, an enzyme that functions for cleaving the amyloid precursor protein to produce Aβ [2]. This excessive Aβ accumulation is believed to trigger a chain reaction of harmful events within the brain cells, including ROS production and dysregulation of glia, particularly microglia and astrocytes. In AD, glia responds to the accumulation of Aβ and contributes to neuroinflammation, amyloid plaque formation, and neuronal damage. Moreover, ROS, produced by glia or neuronal cells in response to Aβ, has been widely recognized for its linkages with mitochondrial dysfunction, cell membrane damage, and neuronal cell death [3–5]. This interconnected pathway highlights the attractive strategies for therapeutic intervention. One promising approach to protect the neurons lies in the attenuation of Aβ-mediated neurotoxicity. Recently, the potential of low-dose BACE1 inhibitors was revealed to lower the levels of Aβ_40_ and Aβ_42_ by 30%, preventing the initial amyloidogenic cleavage and avoiding side effects [6, 7]. Consequently, there is a renewed emphasis on developing more selective but less toxic BACE1 inhibitors used in pre-symptomatic stages in patients with AD. Even BACE1 might not be considered a fundamental player in the progression of AD, nor a key therapeutic target. But BACE1 inhibitor still showed potential as an AD therapeutic agent. Combining with other pharmacological actions could lead to the development of effective neuroprotective agents for preventing AD progression.

The continual increase in AD prevalence indicates the urgent need for the discovery of novel therapeutics. Currently, available drugs are symptomatic drugs incapable of delaying the progression of AD. Despite decades of extensive research efforts, successful disease-modifying medicines for AD remain elusive [1]. Accordingly, current research attention has been paid to naturally occurring antioxidants due to their opportunistic potentials for multidisciplinary therapeutic benefits in alleviating the pathological features associated with AD [8–10]. Naphthoquinone (NQ) derivatives are commonly found in various plants and are associated with defense mechanisms against microbial and insect threats. NQs are well-known for their redox properties, this unique property makes them valuable templates for the discovery of numerous therapeutic agents (i.e., antioxidant, antimicrobial, anticancer, anti-inflammatory, and neuroprotective agents). Amongst others, ANQs are captivating candidates, the substitution of an amino moiety (-NH_2_) on the NQ core could modify the molecular polarity and hydrogen-bonding capacity of the molecules. ANQs have been reported for various biological activities [11–13]. The existing literature highlights several studies indicating that certain NQ derivatives exhibit neuroprotective effects, particularly in the context of oxidative stress and neuroinflammation, key factors involved in the pathogenesis of AD [14–16]. Notably, some NQ derivatives were reported for their abilities to activate SIRT1 protein [17–19]. SIRT1, a protein known as a deacetylase, plays a crucial role in cellular processes (i.e., DNA repair, stress response, and metabolism). SIRT1 is also considered a crucial player in neuroprotection due to its ability to enhance neuronal survival and functions [20–22]. Accordingly, ANQ is an attractive scaffold with the potential to tackle the complexity of neurodegenerative disorders.

Fast and effective drug discovery is particularly essential for rapidly evolving diseases or conditions with urgent medical needs. Computational approaches are commonly utilized to accelerate the pipeline and increase the success rate in drug development [23]. Computational tools have emerged as pivotal strategies to expedite the identification of potential target proteins for novel compounds. In silico target prediction could allow fast and effective target identification in the early stages of development [24]. Molecular docking is commonly used to reveal possible binding modes and interactions of the compounds against the target [25], which would be beneficial for future design and optimization of the compounds. Poor pharmacokinetics (drug-likeness) and severe toxicities of the compounds are key factors rendering the failures in drug development, especially the late-stage occurring ones. Hence, the prediction of these properties within an initial phase of development is highly recommended [26].

**Fig. 1:**
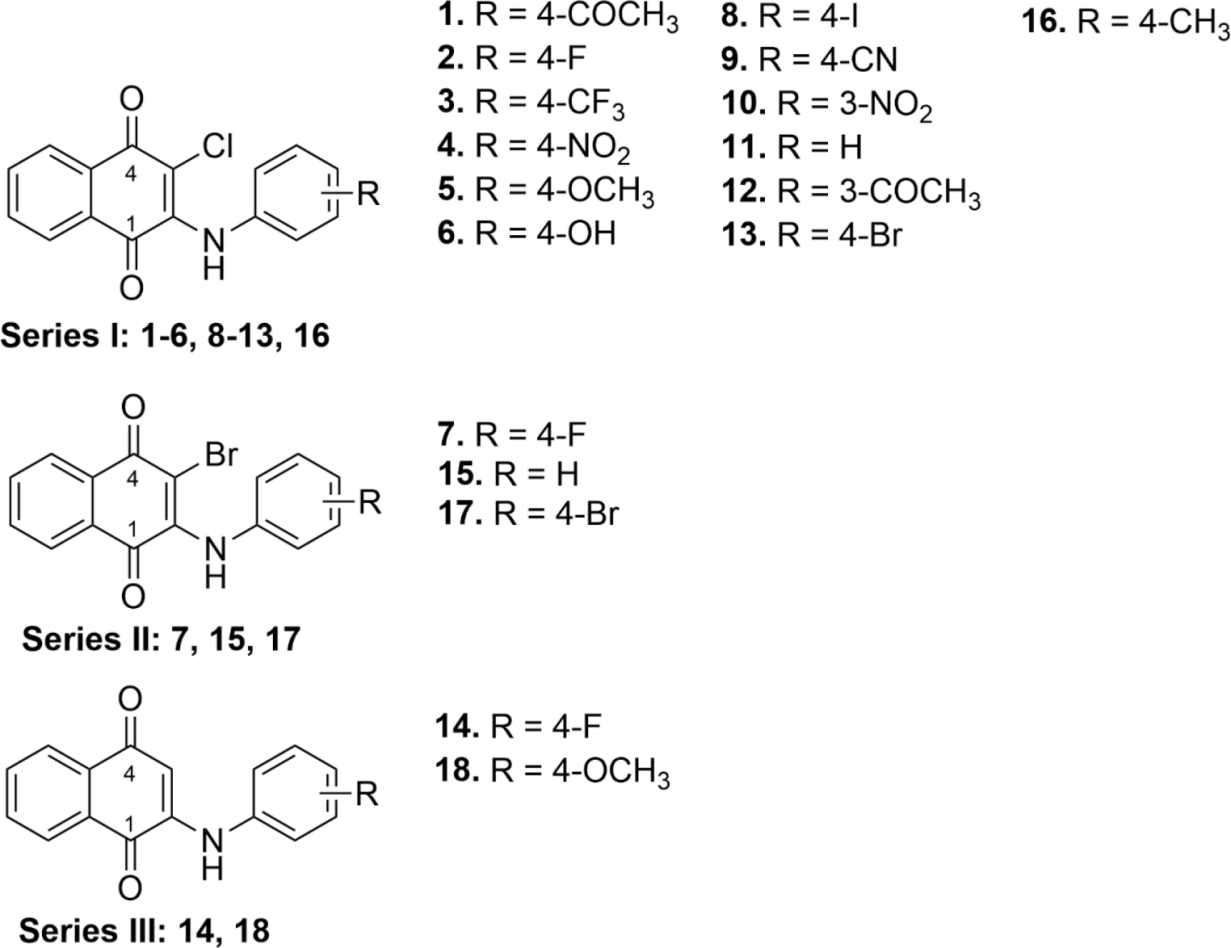
Chemical structures of ANQ derivatives (**1–18**)

In this study, a series of 18 ANQs (Fig. 1) was previously reported by our research group [27, 28]. Some compounds elicited promising anticancer activities, while most of them displayed low cytotoxicity or non-cytotoxicity to normal cell lines. Additionally, various NQ derivatives have been shown to possess neuroprotective effects and SIRT1 activators [17–19]. This promising activity highlights their potentials for therapeutic applications beyond cancer treatment. Herein, these compounds were investigated for their neuroprotective effects against Aβ-induced toxicity in SH-SY5Y cells. Cell viability, catalase-like activity, ROS levels, mitochondrial function, and cell membrane damage as well as SIRT1 and BACE1 activities were assessed. Furthermore, computational studies, (i.e., molecular docking, assessment of pharmacokinetics, and target prediction) were performed to uncover possible binding modes, drug-likeness of the compounds, and potential biological targets.

## Materials and Methods

### Chemical Reagent and Assay

Analytical grade chemicals and reagents were obtained from well-established and reputable suppliers. Beta-amyloid peptide (1–42) (human Aβ_1–__42_, Cat. No. ab120301) was sourced from Abcam (Waltham, Boston, USA). Dulbecco’s Modified Eagle Medium (DMEM, Cat. No. 31600-034), fetal bovine serum (FBS, Cat. No. A5256701), 0.25% trypsin-EDTA (Cat. No. 25200072), and penicillin/streptomycin (Cat. No. 15140122) were procured from Gibco BRL (Gaithersburg, MD, USA). Additionally, 3-(4,5-dimethylthiazol-2-yl)-2,5-diphenyltetrazolium bromide (MTT, Cat. No. M6494) and cell-permeant 2′,7′-dichlorodihydrofluorescein diacetate (DCFDA, Cat. No. D399) were provided by Molecular Probes (Eugene, Oregon, USA). Furthermore, Sigma-Aldrich (St. Louis, Mo, USA) supplied dimethyl sulfoxide (DMSO, Cat. No. 41639), RSV (Cat. No. R5010), mitochondrial-specific fluorescent dye (rhodamine-123, Cat. No. R8004), lactate dehydrogenase (LDH, Cat. No. MAK066), SIRT1 (Cat. No. CS1040), and BACE1 (Cat. No. CS0010) activity assay kits.

### Preparation of Aβ_42_ Oligomer and Investigated Compounds

Aβ_42_ was dissolved in sterile water and incubated at 37 °C for 24 h to facilitate oligomerization. After the incubation period, the solution underwent centrifugation at 14,000 g for 10 min at 4 °C to remove undissolved fibrils. The resulting supernatant, enriched with Aβ oligomers, was meticulously transferred to fresh tubes and stored at 4 °C for subsequent utilization [29, 30].

ANQs (**1**–**18**) were synthesized by nucleophilic substitution or Michael addition of various amines with 1,4-NQs reported by our group [27, 28]. Investigated compounds (**1**–**18**) were individually dissolved in DMSO to generate concentrated stock solutions. These stock solutions were then serially diluted in DMEM supplemented with 10% heat-inactivated FBS and 1% penicillin/streptomycin to achieve the desired working concentrations for the experiment. RSV was employed as a positive control due to its established neuroprotection and SIRT1 activator, as documented in previous literature [31].

### Cell Culture

Human SH-SY5Y neuroblastoma cell line was procured from the American Type Culture Collection (ATCC, VA, USA) and cultivated in 75-cm^2^ flasks. The growth medium consisted of DMEM supplemented with 10% heat-inactivated FBS and 1% penicillin/streptomycin. The culture was maintained at a temperature of 37 °C within a humidified incubator containing 5% CO_2_. To ensure optimal growth, the culture medium was replaced every three days.

Cell subculture was initiated to facilitate subsequent expansion or experimentation. The used medium was aspirated, and the flask underwent a rinsing step with phosphate-buffered saline (PBS). Subsequently, 1 mL of a 0.25% trypsin-EDTA solution was introduced, and the flask was subjected to a minute incubation period at 37 °C to facilitate effective cell detachment. A gentle shaking motion was applied to aid in the dissociation process. Following the trypsinization, 2 mL of complete DMEM was introduced to deactivate the trypsin, and the resulting cell suspension was carefully transferred to a centrifuge tube. Centrifugation at 1,000 rpm for 5 min at 20 °C was performed to precipitate the cells, and the supernatant was subsequently removed. Lastly, the cell pellet was resuspended in fresh complete DMEM and plated into new culture flasks to perpetuate their growth.

### Determination of Cell Viability

The SH-SY5Y neuroblastoma cell line was employed as an experimental model. The cells were initially seeded at a density of 1.0 × 10^5^ cells/mL in 96-well plates for initial adherence. Treatment regimens differed amongst groups including (i) the control group remained unexposed to ANQs (**1**–**18**) or Aβ_42_, (ii) the toxin-induced group was exposed to 1 µM Aβ_42_, (iii) the compound-treated group received treatment with ANQs (1–18) at concentrations ranging from 0.1 to 100 µM for 24 h, (IV) the compound-pretreated group received pretreatment with ANQs (**1**–**18**) at concentrations ranging from 0.1 to 100 µM for 3 h, followed by media refreshment and exposure to 1 µM Aβ_42_ for an additional 24 h. Following the incubation period, 5 mg/mL MTT solution was introduced, and the cells were incubated in darkness at 37 °C for 3 h. Subsequent steps involved media removal, formazan crystal solubilization with 0.04 N HCl in isopropanol, and final absorbance measurement at 570 nm using a microplate reader (Multiskan SkyHigh, Thermo Fisher Scientific, USA). Cell viability was subsequently quantified as a percentage relative to the untreated control group [32].

### Evaluation of Cell Morphology

After the overnight culture at a density of 1.0 × 10^5^ cells/mL on Petri dishes, the SH-SY5Y cells underwent each specified treatment regimen. Subsequently, cell morphology was examined using an inverted light microscope (Olympus Corporation, Tokyo, Japan) at a 20x magnification with multiple fields. Comprehensive images of both treated and untreated cells were captured for subsequent investigation.

### Assessment of Catalase-Like Activity

The catalase-like activity was assessed by monitoring the reduction in hydrogen peroxide (H_2_O_2_) concentration using a modified method derived from the literature of Rasheed RT, 2020 [33], Costa RO, 2018 [34], and Goldblith SA, 1950 [35]. In brief, either compounds at a concentration of 1 µM or cell lysate after treatment regimen were incubated with 590 mM H_2_O_2_ in 0.05 M PBS at pH 7.0 for 30 min. At the end of the incubation, potassium permanganate (KMnO_4_) and sulfuric acid were introduced to the reaction mixture. The purple color of the KMnO_4_ solution, reacting with the residual H_2_O_2_, was transformed into a colorless manganese sulfate (MnSO_4_) product. The absorbance was immediately kinetically measured at a wavelength of 525 nm over 30 min. The average rate of absorbance change throughout the reaction time was utilized to quantify the concentration of H_2_O_2_. The results were subsequently expressed as a percentage of H_2_O_2_ reductance relative to the control group.

### Determination of Intracellular ROS

The generation of intracellular ROS was evaluated using the DCFDA fluorescent probe, which detects ROS-mediated oxidation and produces green fluorescence. SH-SY5Y cells were initially plated in 96-well plates at a concentration of 1.0 × 10^5^ cells/mL and subjected to the treatment protocol. Following the completion of the treatment regimen, 10 µM DCFDA was introduced and subsequently incubated at 37 °C for 30 min in darkness. The fluorescence intensity was measured using a microplate reader at excitation/emission wavelengths of 495/527 nm. Intracellular ROS production was then expressed as a percentage relative to the control group [36].

### Evaluation of MMP

MMP was assessed using the rhodamine-123 fluorescent probe. SH-SY5Y cells were seeded in 96-well plates at a concentration of 1.0 × 10^5^ cells/mL and subjected to the designated treatment regimen. Following the treatment, cells were incubated with 10 µM rhodamine-123 for 30 min in the dark at 37 °C. Fluorescence intensity (488/525 nm excitation/emission) was measured to assess the MMP and expressed as a percentage of the control group [37].

### Assessment of Cellular Membrane Damage

Following respective treatments, supernatant was collected and the LDH activity assay was performed using a commercially available kit according to the manufacturer’s instructions. Briefly, the assay measures the conversion of lactate to pyruvate by LDH, leading to NADH production and increased absorbance at 450 nm. The LDH activity was then expressed as a percentage relative to the control group.

### Measurement of BACE1 Cleavage Activity

The assessment of BACE1 cleavage activity was conducted utilizing the fluorescent BACE1 activity detection kit, following the manufacturer’s guidelines. SH-SY5Y cells were seeded in 6-well plates at a density of 1.0 × 10^5^ cells/mL and allowed to adhere overnight. Subsequently, the cells were subjected to the designated treatments. Following the treatment, cell lysis was carried out in radioimmunoprecipitation assay buffer (RIPA buffer) supplemented with protease inhibitors at 4 °C for 20 min. Protein concentration was then determined using the Bradford assay. Subsequently, the total protein content for each group was assessed using the BACE1 activity assay, and the resulting fluorescent signals were measured using a microplate reader set to an excitation wavelength of 320 nm and an emission wavelength of 405 nm. BACE1 activity was subsequently expressed as a percentage relative to the control group.

### Evaluation of SIRT1 Deacetylase Activity

The deacetylase activity of SIRT1 was assessed using the SIRT1 assay kit, according to the manufacturer’s instructions. SH-SY5Y cells were seeded in 6-well plates at 1.0 × 10^5^ cells/mL and allowed to adhere overnight. Subsequently, the cells underwent the designated treatments. Cells were lysed in cold PBS supplemented with protease inhibitors at 4 °C for 20 min following post-treatment. Then, protein concentration determination was executed for each cell group using the Bradford assay. Finally, the total amount of protein from each group was determined through the SIRT1 assay, and the resulting fluorescent signals were detected using a microplate reader with excitation and emission wavelengths set at 340 and 445 nm, respectively. SIRT1 activity was then expressed as a percentage relative to the control group.

### Molecular Docking Analysis

Possible binding modes of ANQs against the SIRT1 were simulated using AutoDockTools v.4.2.6 [38]. The crystal structure of human SIRT1, in complex with three RSV molecules and an AMC-containing peptide, was retrieved from the Protein Data Bank (PDB code: 5BTR) at 3.2 Å resolution [39]. Initially, co-crystallized ligands were excluded, and only chain A was selected for further preparation by adding polar hydrogens and charges. Docking parameters were set to be a grid spacing of 0.375 Å and a grid box size of 50 × 50 × 50 points. The grid box center was positioned at -23.315, 65.890, and 14.723 to encompass the entire activator-binding site between the C-terminal and N-terminal domains of SIRT1. Rotational bonds were defined as rigid for the protein, but flexible for the investigated compounds. Each docking simulation involved 100 independent runs with the Lamarckian Genetic Algorithm as the search strategy and a medium maximum energy level [40]. The accuracy of the docking simulations was confirmed by calculating the root-mean-square deviation (RMSD) between the co-crystallized and re-docked RSV molecules. Binding interactions and key residues were visualized and analyzed using Discovery Studio Visualizer 2016 (BIOVIA, Dassault Systèmes, USA).

### In Silico Assessment of Pharmacokinetics and Drug-Like Properties

To evaluate the drug-likeness and potential toxicities of the investigated compounds, chemical structures in SMILES format were employed for the prediction using the web server tools, including SwissADME (http://www.swissadme.ch/) [41], pkCSM (http://biosig.unimelb.edu.au/pkcsm/) [42], and ProTox-II (http://tox-new.charite.de/protox_II/) [43]. Then, the data received from all web servers were analyzed.

### Target Prediction

For the prediction of AD-related gene targets pertaining to our investigated compounds, AD-related genes were sourced from two prominent databases, including DisGeNET [44] with a score cutoff of 0.2 and GeneCards [45] with a score threshold of 2.57, calculated through median determination. Subsequently, the possible protein targets of the investigated compounds were predicted using two well-established web-based servers including SuperPred [46] and SwissTargetPrediction [47]. Stringent criteria were set, requiring a minimum cutoff of possibility exceeding 60% and accuracy surpassing 75% for SuperPred. Additionally, proteins with a probability of 0 were eliminated from SwissTargetPrediction, ensuring the reliability of the predictions. The shared common targets were visualized as an intersected area using the VENNY 2.1 online tool (https://bioinfogp.cnb.csic.es/tools/venny/*)* to identify a refined set of potential AD-associated targets specifically interacting with our compounds. This intersection represents a focused list of the target proteins with a high likelihood of being involved in AD as well as a high potential to interact with the studied compounds.

To further investigate the network of interactions and identify key players, the STRING database [48] and Cytoscape software [49] were utilized for visualization. A protein-protein interaction network was constructed with a high confidence level set at 0.9 and analyzed within Cytoscape software. Proteins with a degree of zero (no connections) were excluded. The prediction focused on only well-connected nodes within the network. Then, the nodes were ranked based on three measures (i.e., degree, closeness centrality, and betweenness centrality) to prioritize the most influential proteins within the network. These metrics capture the direct connections, information flow, and bridging potential of each protein.

### Statistical Analysis

Statistical analyses were performed using GraphPad Prism 6 (GraphPad Software Inc., CA, USA). One-way analysis of variance (ANOVA) was utilized for statistical comparisons between groups, followed by a Tukey–Kramer post-hoc test. The results are expressed as mean ± standard error of the mean (SEM) derived from three or more independent experiments. A probability (*P*) value less than 0.05 was considered statistically significant. All raw data are provided in the supplementary file.

## Results

### Synthesis of ANQs (1–18)

The chemical structures of 18 ANQ derivatives (**1–18**, Fig. 1) were previously reported by our group [27, 28] including series I (**1**–**6**, **8**–**13**, **16**), series II (**7**, **15**, **17**), and series III (**14**, **18**). These compounds possess a 1,4-NQ core structure with 2-amino or 2-amino-3-halo substituents, in which the 2-amino was substituted by phenyl ring bearing R (electron withdrawing/electron donating groups) substituents on 3- or 4-position.

### ANQs Enhance Cell Viability Against Aβ_42_-Treated Cells

**Fig. 2:**
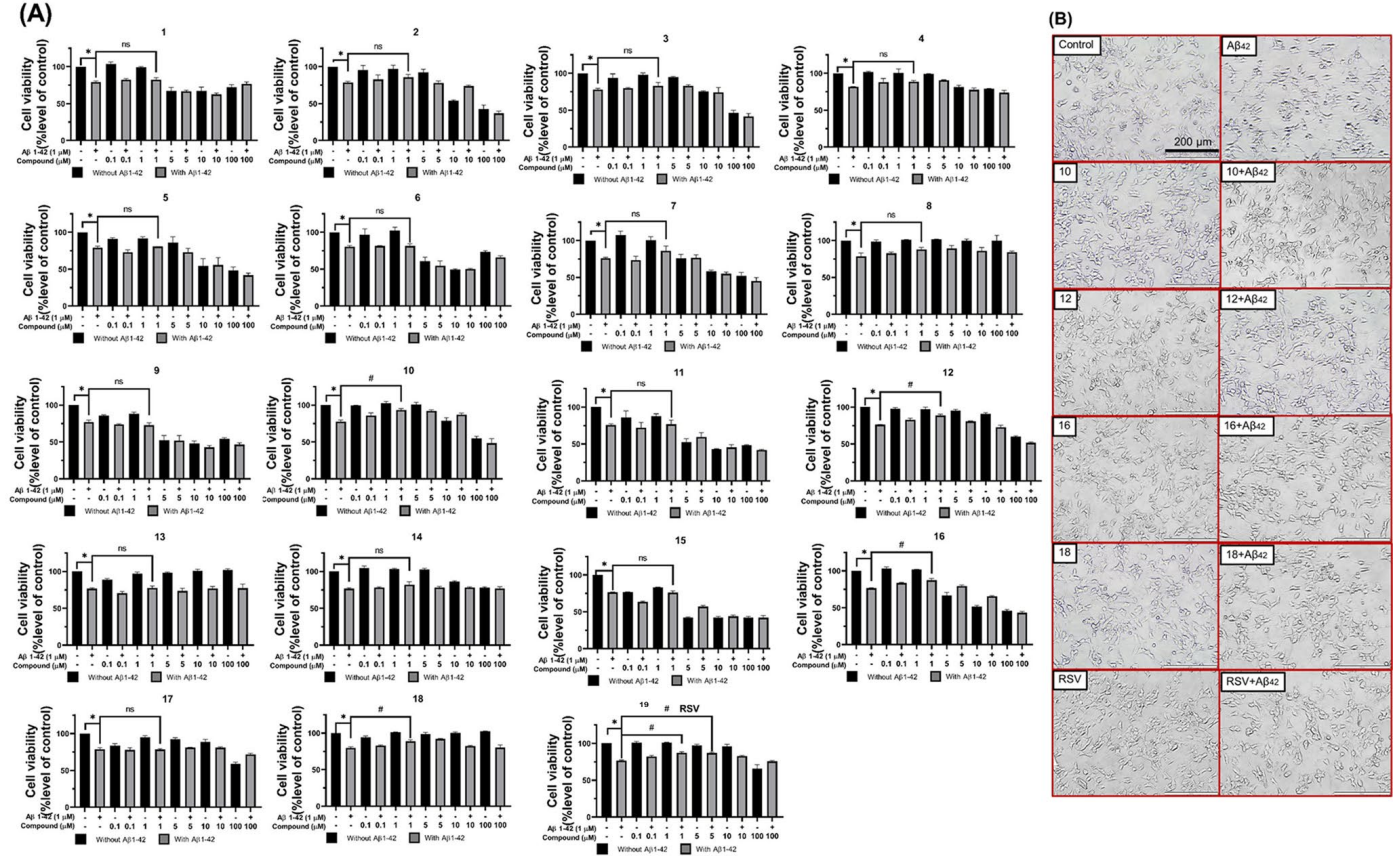
Effects of 18 ANQ derivatives on SH-SY5Y neuroblastoma cell viability and morphology. (**A**) Cell viability was represented as a percentage relative to the control group. *P < 0.05 compared to the control; #*P* < 0.05 compared to the Aβ_42_. (**B**) Morphological alterations of SH-SY5Y cells were observed under the inverted light microscope at ×20 magnification. Scale bar = 200 μm

The effect of ANQ derivatives on SH-SY5Y neuroblastoma cell viability was evaluated using the MTT assay. SH-SY5Y cells were pretreated with various concentrations of compounds (0.1–100 µM) for 24 h in the absence of Aβ_42_. The results indicated that all compounds exhibited no significant effect on cell viability at minimal doses (0.1–1 µM). However, some compounds demonstrated toxicity to the cells at higher concentrations (5–100 µM). To represent the AD model, the toxicity of 1 µM Aβ_42_ alone on SH-SY5Y cells was investigated and revealed a notable 25% reduction in cell viability upon the treatment (Fig. 2A).

The neuroprotective potential of ANQ derivatives was explored by pretreating the SH-SY5Y cells with each compound for 3 h before exposure to 1 µM Aβ_42_ for an additional 24 h. Results demonstrated that 1 µM compounds significantly enhanced cell viability of the pretreated cells, specifically compound **10** (93.38 ± 1.69%), compound **12** (88.66 ± 1.54%), compound **16** (87.38 ± 2.32%), and compound **18** (88.80 ± 2.12%), compared to the cells treated with Aβ_42_ alone (Fig. 2A). Notably, these compounds elicited protective effects comparable to that of the RSV, a reference compound known for its cell viability protection (87.20 ± 1.13%). Based on the results, these four ANQs (**10**, **12**, **16**, and **18**) at a concentration of 1 µM were selected for further explorations to elucidate potential neuroprotective mechanisms.

### ANQs Did Not Alter Cellular Morphology

Bright-field microscopy revealed the protective effects of the selected compounds (**10**, **12**, **16**, and **18**) against Aβ_42_-induced neurotoxicity in SH-SY5Y cells. Exposure to Aβ_42_ alone caused notable morphological changes, including cell shrinkage, detachment, and rounding (Fig. 2B). However, pretreatment with any of the selected ANQs or RSV at 1 µM for 3 h significantly mitigated these Aβ_42_-induced alterations. The pretreated cells displayed minimized damage, improved adherence, and normal growth patterns compared to the Aβ_42_-exposed group (Fig. 2B). These findings suggested that the selected ANQs hold the potential for protecting the neuronal morphology against Aβ_42_-induced neurotoxicity.

### Catalase-Like Activity Increased by ANQs Pretreatment

**Fig. 3:**
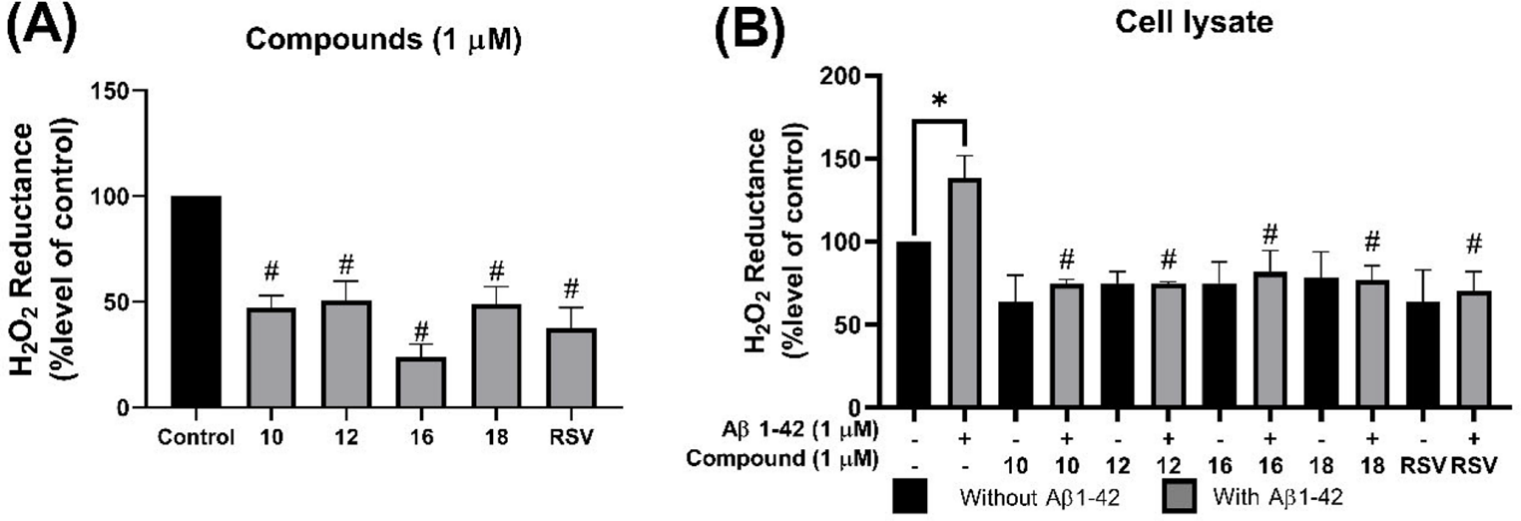
Effects of selected ANQs (**10**, **12**, **16**, and **18**) on catalase-like activity. (**A**) Percentage of H_2_O_2_ reductance from selected compounds (1 µM). (**B**) Percentage of H_2_O_2_ reductance from post-treatment cell lysate. *P < 0.05 compared to the control cells; #*P* < 0.05 compared to the Aβ_42_ group

The antioxidant potential of the selected ANQs (**10**, **12**, **16**, and **18**) at 1 µM was evaluated based on their abilities to quench H_2_O_2_. A redox reaction between KMnO_4_ and H_2_O_2_, resulting in its decolorization, was employed to monitor the H_2_O_2_ levels. Particularly, the co-incubation of H_2_O_2_ with these compounds significantly decreased the reduction rate of KMnO_4_ by 70% compared to that of the H_2_O_2_ alone (Fig. 3A).

Furthermore, the antioxidant effect of these compounds on the induction of catalase-like activity was investigated in cell lysates following the designated treatments. Interestingly, pretreatment with the tested compounds significantly increased catalase-like activity compared to the cells solely exposed to 1 µM Aβ_42_ for 24 h, as observed in the decreased H_2_O_2_ concentration after incubating with the cell lysate (Fig. 3B). This suggested that the ANQs might enhance the intrinsic antioxidant defense mechanisms of the cells.

### ANQs Diminished Intracellular ROS Generation Against Aβ_42_-Treated Cells

**Fig. 4:**
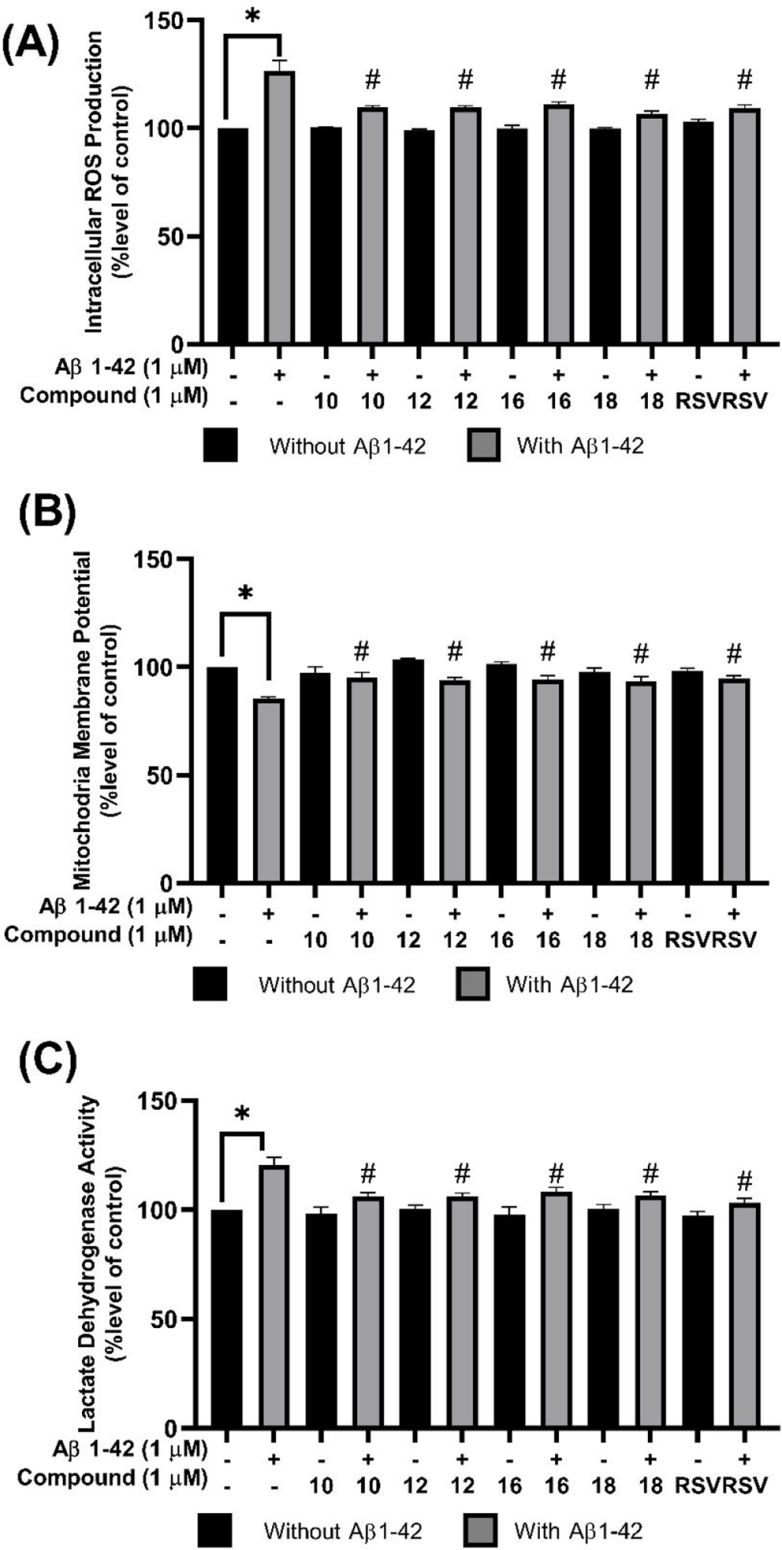
Protective effects of selected ANQs (**10**, **12**, **16**, and **18**) at 1 µM against Aβ_42_-induced pathological alterations in SH-SY5Y cells. (**A**) Intracellular ROS production, (**B**) MMP, and (**C**) LDH activity. *P < 0.05 compared to the control; #*P* < 0.05 compared to the Aβ_42_ treatment

The effect of selected compounds (**10**, **12**, **16**, and **18**) on intracellular ROS generation was evaluated using the DCFDA fluorescent probe. The cells treated with 1 µM Aβ_42_ for 24 h exhibited a notable increase in fluorescence intensity reaching 126.57 ± 4.76% compared to the control (Fig. 4A). Conversely, pretreatment with 1 µM of compounds **10**, **12**, **16**, **18**, or RSV for 3 h prior to Aβ_42_ exposure significantly reduced ROS accumulation. This was evident by a decrease in DCFDA fluorescence intensity compared to the control group (compounds **10**: 109.70 ± 0.74%, **12**: 109.54 ± 0.95%, **16**: 111.16 ± 0.97%, **18**: 106.87 ± 1.13%, and RSV: 109.22 ± 1.65%). These findings suggested that the selected ANQs (**10**, **12**, **16**, and **18**) potentially decrease intracellular ROS production comparable to that of the RSV against Aβ_42_-induced pathogenesis in SH-SY5Y cells. While this result provides preliminary evidence for the potential antioxidant activity of the ANQs, further complementary investigations are still required to fully assess the antioxidant potency of the ANQ derivatives.

### MMP Maintained by ANQs Pretreatment

The protective potential of selected compounds (**10**, **12**, **16**, and **18**) against mitochondrial dysfunction was investigated by assessing the fluorescent signal emitted from the rhodamine-123 dye. It was found that exposure to 1 µM Aβ_42_ for 24 h resulted in a significant reduction in MMP (85.26 ± 0.91% lower than that of the control group, Fig. 4B). Nevertheless, pretreatment with 1 µM of compounds **10**, **12**, **16**, **18**, or RSV significantly preserved the MMP by 95.24 ± 2.32%, 94.05 ± 1.20%, 94.41 ± 1.77%, 93.52 ± 2.01%, and 94.94 ± 1.09%, respectively, compared to the control group. This suggested the remarkable ability of the compounds to safeguard the mitochondrial function from Aβ_42_-mediated neurotoxicity.

### ANQs Protected Cell Membrane Damage in Aβ_42_-Induced LDH Release

The cell membrane damage was assessed by the release of LDH. Exposure to 1 µM Aβ_42_ for 24 h significantly increased the release of LDH to 120.61 ± 3.48% compared to the control cells (Fig. 4C). Importantly, pretreatment with 1 µM of tested compounds (**10**, **12**, **16**, and **18**) or RSV significantly counteracted this Aβ_42_-induced LDH release as observed by the reduction in LDH release to near-control levels (compounds **10**: 106.24 ± 1.66%, **12**: 106.36 ± 1.29%, **16**: 108.44 ± 1.89%, **18**: 106.73 ± 1.62%, and RSV: 103.35 ± 1.95%). The results showed the membrane-protective properties of these ANQs, which could potentially contribute to their neuroprotective abilities.

### ANQs Mitigated BACE1 Cleavage Activity in Aβ_42_-Induced SH-SY5Y Cells

**Fig. 5:**
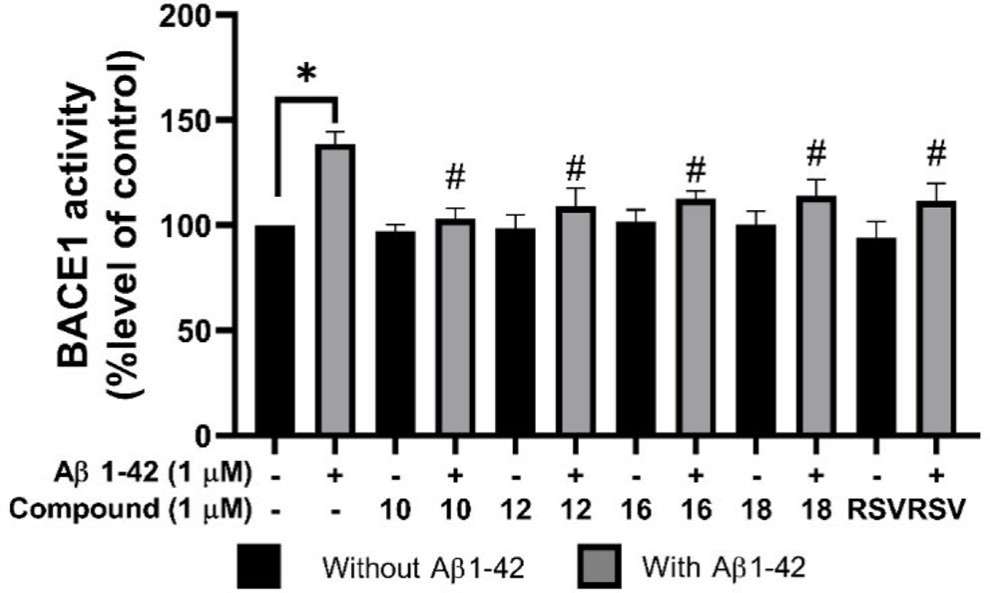
Effects of selected ANQs (**10**, **12**, **16**, and **18**) at 1 µM on BACE1 activity against Aβ_42_-induced SH-SY5Y cells. *P < 0.05 compared to the control; #*P* < 0.05 compared to the Aβ_42_ group

The influence of selected ANQ derivatives (**10**, **12**, **16**, and **18**) on BACE1 activity within SH-SY5Y cells was assessed using the BACE1 activity kit. Upon treatment with 1 µM Aβ_42_ for 24 h, there was a significant increase in intracellular BACE1 activity (138.81 ± 5.71% compared to the control group, Fig. 5). However, pretreatment with the selected ANQs (1 µM) for 3 h prior to 1 µM Aβ_42_ exposure resulted in a notable reduction in BACE1 activity (**10**: 103.05 ± 5.22%, **12**: 108.93 ± 8.81%, **16**: 112.53 ± 3.69%, and **18**: 113.95 ± 7.83%) compared to the control group. Interestingly, the compounds showed comparable effects with those observed for RSV, which maintained BACE1 activity at 111.67 ± 8.16%. These findings highlighted the potential of the selected ANQs in mitigating BACE1 activity.

### SIRT1 Deacetylase Activity Modulated by ANQs Pretreatment

**Fig. 6:**
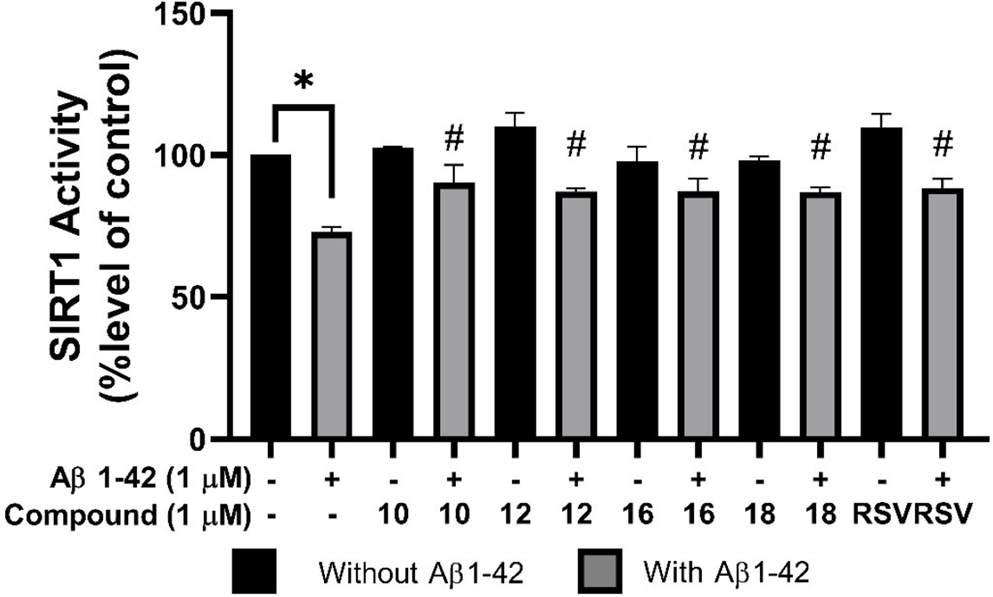
Effects of selected ANQs (**10**, **12**, **16**, and **18**) at 1 µM on SIRT1 activity in Aβ_42_-induced SH-SY5Y cells. *P < 0.05 compared to the control; #*P* < 0.05 compared to the Aβ_42_ group

The influence of the chosen ANQs (**10**, **12**, **16**, and **18**) on SIRT1 activity within SH-SY5Y cells was examined using the SIRT1 activity kit. Treatment with 1 µM Aβ_42_ for 24 h significantly reduced SIRT1 activity to 72.93 ± 1.61% compared to the control group (Fig. 6). This indicated the potential role of SIRT1 in Aβ_42_-mediated neurodegeneration. However, pretreatments with ANQs **10**, **12**, **16**, and **18** (1 µM) for 3 h before Aβ_42_ exposure remarkably preserved SIRT1 activity by 90.34 ± 6.17%, 87.08 ± 1.05%, 87.19 ± 4.56%, and 86.93 ± 1.75%, respectively, in comparison to the control group. The compounds showed similar effects with those observed for RSV, a well-known SIRT1 activator, which exhibited sustained SIRT1 activity up to 88.32 ± 3.23%. These findings underscored the potential of selected ANQs in preserving SIRT1 activity.

### Binding Interaction of ANQs in SIRT1

Molecular docking was conducted to elucidate potential binding modes of the selected compounds (**10**, **12**, **16**, and **18**) toward the target protein, SIRT1 (PDB code 5BTR). This protein structure was selected due to its sufficient resolution and the co-crystallized RSV, a well-known SIRT1 activator, which was used as a reference compound for validation of docking protocol to provide reliable details and binding characteristics necessary for accurate molecular docking. To validate the reliability of simulations, redocking of the co-crystalized RSVs was initially performed. The calculated mean binding free energies for the three co-crystallized molecules (RSV1, RSV2, and RSV3) were determined to be − 7.49, − 7.51, and − 7.39 kcal/mol, respectively. The calculated RMSD value below 2.0 was obtained, indicating the preferable accuracy of the docking protocol. Subsequently, the validated protocol was utilized to explore the binding modes of the studied compounds.

**Fig. 7:**
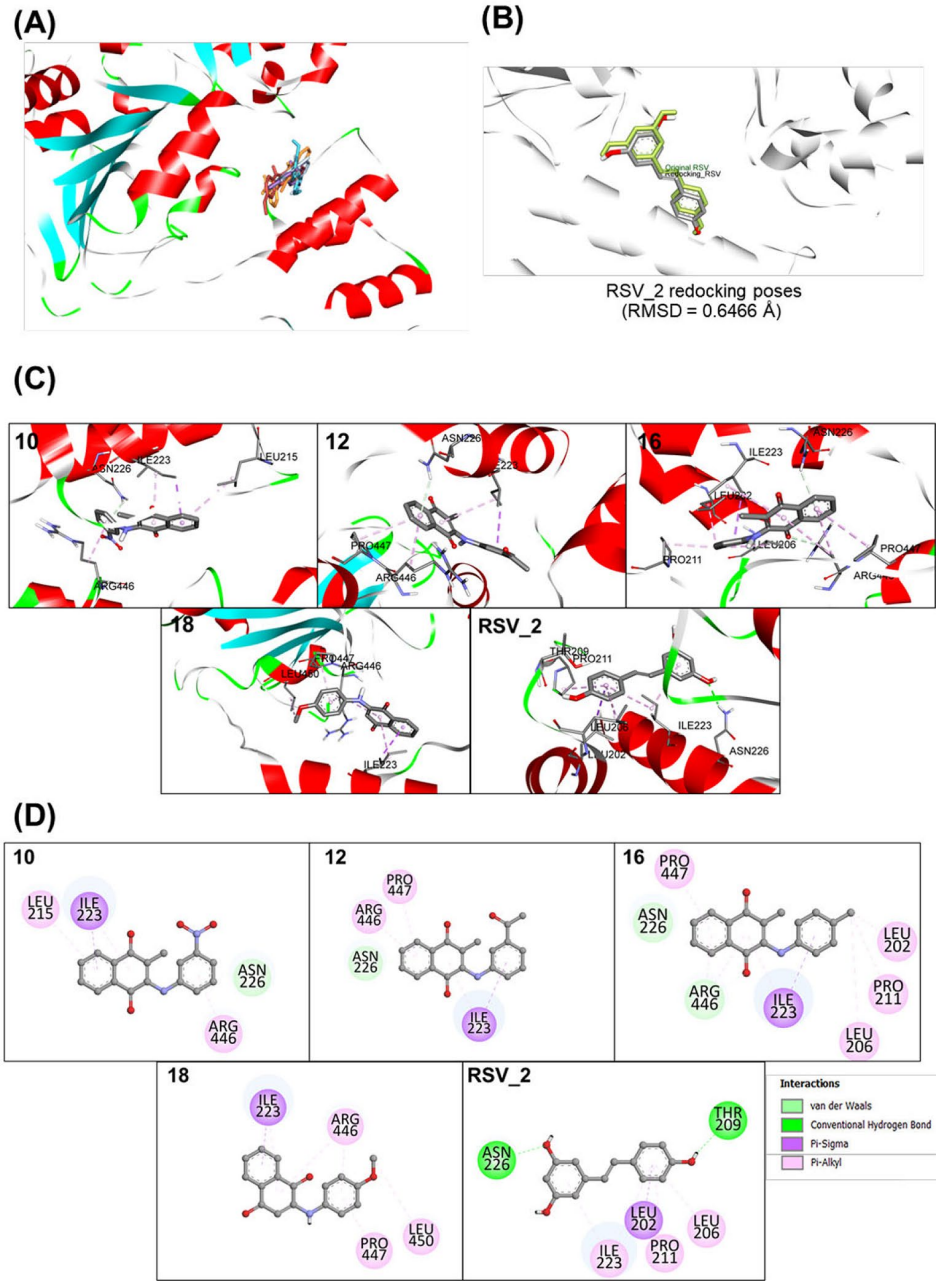
Possible binding modes and ligand-protein interactions of the selected ANQs (**10**, **12**, **16**, and **18**) against the SIRT1 activator-binding site. (**A**) Three-dimensional (3D) representations of the crystal structure of SIRT1 (PDB: 5BTR) bound with RSV and all studied compounds. (**B**) 3D poses of redocking RSV with RMSD of 0.6466 Å. (**C**) 3D depictions of SIRT1 binding interactions with RSV and individual compounds within the activator-binding site. (**D**) Two-dimensional (2D) ligand-protein interaction diagrams of RSV and studied compounds. The interacting residues are depicted as colored circles based on the type of interaction

Docking simulations of the studied compounds showed that all investigated compounds (**10**, **12**, **16**, and **18**) could suitably bind within the same activator-binding site of SIRT1 in similar manners to those of RSVs, Fig. 7A-C. Notably, the binding free energies of the docked ANQs **10**, **12**, **16**, and **18** (− 9.91, − 10.47, − 9.49, and − 9.34 kcal/mol, respectively) were found to be more favorable than those of the RSV cluster molecules. However, docking algorithms often favor larger compounds because they can form more atomic interactions within the binding site, leading to molecular weight bias in the docking scores. Since our compounds are larger than RSV, this bias could result in an overemphasis on their binding affinities. To address this, we evaluated additional factors such as interacted amino acid and type of interactions to ensure that the observed binding affinities are not solely a result of molecular size but reflect interactions between the compounds and SIRT1. Furthermore, the results of SIRT1 activity assay corroborates these findings, confirming the functional significance of these interactions.

To reveal the pivotal binding interactions, two-dimensional ligand-protein interaction diagrams were generated (Fig. 7D). Among three RSV molecules, RSV2 served as a reference model due to its lowest binding free energy among others and its close spatial proximity to the studied compounds. It was demonstrated that the binding of RSV2 was primarily governed by the formations of pi-alkyl interactions with residues LEU206, PRO211, and ILE223, along with a hydrogen bond formation with THR209 and ASN226, as well as a pi-sigma interaction with LEU202. The studied compounds were found to share some key interacting amino acid residues with RSV2 (i.e., ILE223 and ASN226). Notably, the NQ core skeleton and the substituted phenyl ring presented in the compounds could mimic the two phenyl rings of the RSV by playing parts in the formations of pi-alkyl and pi-sigma interactions. However, conventional hydrogen bonding with THR209 residue observed in the binding of RSV was not observed in any studied compounds.

### In Silico Predictions of Pharmacokinetics and Drug-Likeness of ANQs

A comprehensive prediction of pharmacokinetics of the selected compounds (**10**, **12**, **16**, and **18**) was conducted employing three established web-based tools (i.e., SwissADME, pkCSM, and ProTox-II) as provided in Table 1. The results indicated that all compounds are drug-like molecules, as shown by the criteria of Lipinski’s, Veber’s, and Ghose’s rules. The compounds exhibited favorable water solubility, optimal lipophilicity (log *P* < 5), as well as high intestinal absorption (91.92–94.58%) for oral administration. Additionally, these compounds provided moderate drug distribution potential (human VDss of 0.15–0.23 log L/kg) as well as moderate CNS and BBB permeabilities, which suggested their abilities to reach the target site in the CNS.

For metabolic considerations, all compounds were identified as substrates of CYP3A4, a major cytochrome P450 enzyme responsible for metabolizing over 90% of available drugs. Furthermore, they were predicted as inhibitors of CYP1A2, CYP2C19, or CYP2C9, which implicated an alternative metabolic fate and potential drug-drug interactions. These findings warrant further investigations regarding their metabolic profiles and possible interactions with other medications or substances.

In terms of drug elimination and toxicity predictions, the studied compounds were predicted to exhibit total clearance ranging from − 0.018 to − 0.186 log mL/min/kg. All of them, except for compound **12**, were also identified as substrates of the renal OCT2. This issue should be further investigated to reveal their potential pharmacokinetics-related drug-drug interaction potential. Regarding toxicity, all compounds were classified as moderately hazardous with potential for hepatotoxicity, carcinogenicity, immunogenicity, and mutagenicity. These predictions highlighted the need for further in vitro and in vivo studies to comprehensively assess their safety profiles.

**Table 1:** Predictions of physicochemical characteristics, pharmacokinetics, and toxicity profiles of the selected compounds (**10**, **12**, **16**, and **18**)^a^

Compound	10	12	16	18
Physicochemical Properties				
Formula	C_16_H_9_ClN_2_O_4_	C_18_H_12_ClNO_3_	C_17_H_12_ClNO_2_	C_17_H_13_NO_3_
Molecular weight (g/mol)	328.71	325.75	297.74	279.29
Num. heavy atoms	23	23	21	21
Num. arom. heavy atoms	12	12	12	12
Fraction Csp3	0	0.06	0.06	0.06
Num. rotatable bonds	3	3	2	3
Num. H-bond acceptors	4	3	2	3
Num. H-bond donors	1	1	1	1
Molar refractivity	86.69	88.06	82.83	79.56
TPSA (Å²)	91.99	63.24	46.17	55.40
Log Po/w (iLOGP)	1.76	2.13	2.37	2.53
Water solubility	Moderately soluble	Moderately soluble	Moderately soluble	Soluble
**Drug-likeness**				
Lipinski	Yes; 0 violation	Yes; 0 violation	Yes; 0 violation	Yes; 0 violation
Ghose	Yes	Yes	Yes	Yes
Veber	Yes	Yes	Yes	Yes
**Pharmacokinetics**				
**Absorption**				
CaCO_2_ permeability	0.93	0.99	1.38	1.31
Intestinal absorption	91.92	94.58	93.02	94.44
GI absorption	High	High	High	High
**Distribution**				
VDss^b^ (human)	0.15	0.20	0.23	0.15
Low < -0.15, high > 0.45	Moderate	Moderate	Moderate	Moderate
BBB^c^ permeability	-0.28	-0.08	0.15	0.05
Low < -1, high > 0.3	Moderate	Moderate	Moderate	Moderate
CNS^d^ permeability	-1.96	-1.88	-0.81	-2.01
Low < -3, high >-2	Pass	Pass	Pass	Moderate
**Metabolism**				
CYP2D6 substrate	No	No	No	No
CYP3A4 substrate	Yes	Yes	Yes	Yes
CYP1A2 inhibitor	Yes	Yes	Yes	Yes
CYP2C19 inhibitor	Yes	Yes	Yes	Yes
CYP2C9 inhibitor	Yes	Yes	No	No
CYP2D6 inhibitor	No	No	No	No
CYP3A4 inhibitor	No	No	No	No
**Elimination**				
Total Clearance	0.13	-0.02	-0.01	0.19
Renal OCT2^e^ substrate	No	Yes	Yes	Yes
**Toxicity**				
Predicted LD_50_^f^ (mg/kg)	2000	2000	1260	2000
Predicted Toxicity	Class: 4	Class: 4	Class: 4	Class: 4
Hepatotoxicity	Inactive	Active	Active	Active
Carcinogenicity	Inactive	Inactive	Active	Active
Immunotoxicity	Active	Active	Inactive	Active
Mutagenicity	Active	Active	Active	Active
Cytotoxicity	Inactive	Inactive	Inactive	Inactive

^a^The prediction was performed using SwissADME (http://www.swissadme.ch/), pkCSM (http://biosig.unimelb.edu.au/pkcsm/), and ProTox-II (http://tox-new.charite.de/protox_II)

^b^VDss, volume of distribution

^c^BBB, blood–brainbarrier

^d^CNS, central nervous system

^e^Renal OCT2, renal organic cation transporter 2

^f^LD_50_, 50% lethal dose

### Target Prediction of ANQs with Neuroprotection

**Fig. 8:**
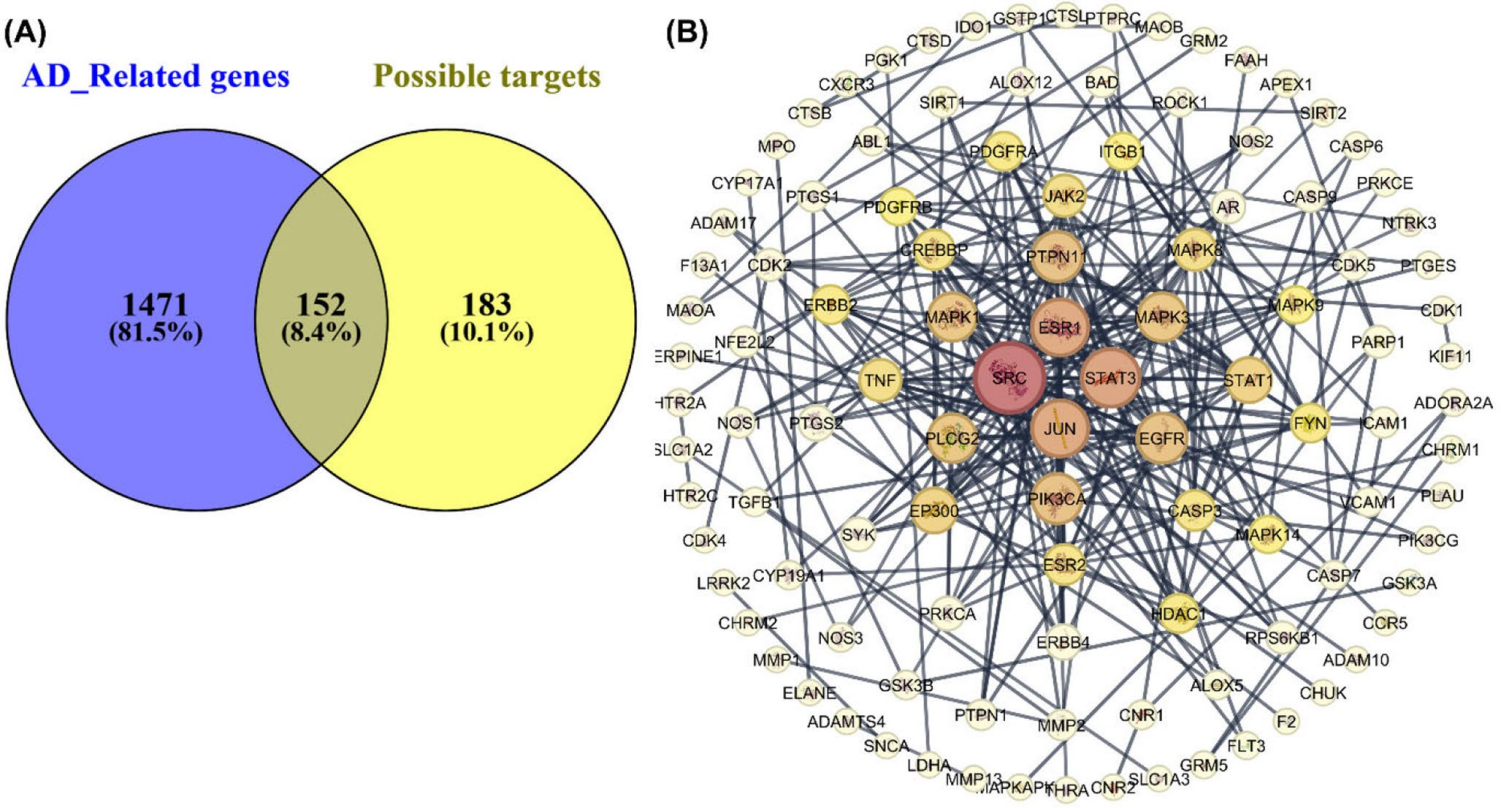
Target prediction and core target analysis of selected ANQs (**10**, **12**, **16**, and **18**) in AD. (**A**) Venn diagram of the intersection between AD-related genes and predicted targets of compounds. (**B**) Protein-protein interaction network of 108 core targets

Diverse resources were employed to comprehensively identify potential therapeutic targets of the studied compounds playing roles in AD. A catalog of 1,623 disease-associating genes was collected. A library of 335 potential protein targets for our investigated compounds was identified. Analysis of overlapping targets revealed a set of 152 shared common genes between the AD-related list and the compound’s predicted targets, Fig. 8A. This finding indicated that our ANQs could potentially interact with crucial proteins implicated in AD pathogenesis. A protein-protein interaction network was created to further explore the relationships among these identified targets resulting in a set of 108 proteins with high connections (Fig. 8B). These proteins were subsequently ranked using centrality measures (i.e., degree, closeness centrality, and betweenness centrality) to identify potential protein hubs within the network. A set of top 20 ranked core target proteins are summarized in Table 2. This ranking prioritizes proteins that play key roles in the interaction network. According to our analysis, SRC, STAT, and JUN emerged as the top 3 nodes based on their centrality measures. This suggests that these proteins are highly connected and influential within the network, potentially playing pivotal roles in the underlying biological processes. While our analysis provides a valuable overview of the protein-protein interaction network, it is important to acknowledge its limitations. The completeness of the pathways represented in the network depends on the quality and comprehensiveness of the underlying used databases. Moreover, this is just a prediction and pathway in the network might not be fully represented, since non-targeted proteins were not use to construct the network. This could potentially overlook important interactions and regulatory mechanisms that are critical to understand the complete biological context. Notably, many well-established players in AD are included in this top-ranked list, which supports the potential therapeutic relevance of our findings.

**Table 2:** List of top 20 ranked core target proteins^g^

Protein	Full Name	Involvement in AD	Expression Level Change in AD	Potential Drug Target	Degree	Closeness Centrality	Betweenness Centrality
SRC	Proto-oncogene tyrosine-protein kinase Src	Essential for the signaling response induced by Aβ and subsequent elevations in neuroinflammation in AD [50, 51].	Increase	Inhibitor	28	0.540	0.207
STAT3	Signal transducer and activator of transcription 3	Neuroinflammation and impaired neuronal function [52].	Increase	Inhibitor	21	0.517	0.169
JUN	c-Jun	Impaired neuronal function, tau phosphorylation, and Aβ production [53].	Increase	Inhibitor	20	0.503	0.159
ESR1	Estrogen receptor 1	Neuroprotective effects in females [54].	Decrease (females)	Activator	20	0.487	0.093
EGFR	Epidermal growth factor receptor	Amyloidogenic receptor facilitates the cellular uptake of preformed fibril and promotes the seeding of misfolded proteins [55].	Increase	Inhibitor	16	0.436	0.026
PTPN11	Protein tyrosine phosphatase, non-receptor type 11	May induce aggregation of Aβ, ER stress, and apoptosis [56, 57]	Increase	Inhibitor	16	0.441	0.021
PIK3CA	Phosphatidylinositol-4,5-bisphosphate 3-kinase catalytic subunit alpha	May reduce hyper-phosphorylation of Tau and inhibit apoptosis [58, 59].	Decrease (Varied)	Activator	16	0.419	0.046
MAPK3	Mitogen-activated protein kinase 3 (ERK1)	Impaired memory and learning, as well as pro-inflammatory activation of microglia [60, 61].	Increase	Inhibitor	15	0.472	0.066
PLCG2	Phospholipase C gamma 2	May contribute to neurodegeneration [62, 63].	Increase activity	Inhibitor	15	0.417	0.059
MAPK1	Mitogen-activated protein kinase 1 (ERK2)	May contribute to neurodegeneration, inflammation, and apoptosis [64, 65].	Increase activity	Inhibitor	15	0.457	0.060
STAT1	Signal transducer and activator of transcription 1	Neuroinflammation and impaired neuronal function [66].	Increase	Inhibitor	13	0.455	0.015
EP300	E1A binding protein p300	Regulates gene expression and potential role in AD progression [67].	Increase	Inhibitor	13	0.444	0.098
JAK2	Janus kinase 2	Associated with Tau-induced neurodegeneration [53, 68].	Increase activity	Inhibitor	11	0.399	0.003
TNF	Tumor Necrosis Factor	Neuroinflammation and neuronal death [69].	Increase	Inhibitor	11	0.466	0.156
MAPK8	Mitogen-activated protein kinase 8 (JNK)	Associated with Tau-induced neurodegeneration [53, 68].	Increase activity	Inhibitor	11	0.441	0.046
ESR2	Estrogen receptor 2	Potential role in AD, less studied than ESR1 [70]	Unknown	N/A	10	0.419	0.016
CASP3	Caspase 3	Neuronal cell death [71, 72].	Increase	Inhibitor	10	0.387	0.084
CREBBP	CREB-binding protein	Potential role in memory and learning, role in AD is unclear [73].	Decrease	Activation	10	0.436	0.045
HDAC1	Histone deacetylase 1	Maintaining genomic integrity [74].	Decrease	Activation	9	0.417	0.025
ERBB2	Erb-B2 receptor tyrosine kinase 2 (HER2)	Impaired lysosomal degradation results from suppressed autophagic flux [75].	Increase	Inhibitor	9	0.405	0.002

^g^A high confidence level set at 0.9 and analyzed within Cytoscape software. Proteins with a degree of zero (no connections) were excluded

## Discussion

In this study, a series of 18 ANQ derivatives (**1**–**18**) was comprehensively investigated for neuroprotective potentials to reveal their multifaceted effects in combating Aβ_42_-induced cellular damage in SH-SY5Y cells. Aβ_42_ is well-known for its ability to mimic key pathological features of AD (i.e., cellular toxicity and neuroinflammation). This creates a highly relevant model system for investigating the mechanisms underlying the disease and evaluating potential therapeutic interventions [76]. Previous studies reported that 1 µM of Aβ_42_ effectively induces AD pathology and diminishes cell viability as much as 70%. Accordingly, Aβ_42_ at 1 µM was selected as the neurotoxic agent to induce the AD studied model using the cell culture without exposure to Aβ_42_ as the control group.

Cell viability indicated that the tested compounds elicited a dose-dependent effect on neuronal cell survival. All compounds are non-cytotoxic at low concentrations (0.1–1 µM), but some of them showed toxicity at higher concentrations (5–100 µM). Thus, a concentration of 1 µM was selected for further investigations. It was shown that pretreatment with 1 µM of compounds (**1**–**18**) enhanced cell viability of the Aβ_42_-induced cells. However, only four compounds (**10**, **12**, **16**, and **18**) demonstrated promising protective effects comparable to that of the RSV (Fig. 2A) and were selected for further experiments. The selected compounds (**10**, **12**, **16**, and **18**) effectively protected the cells against Aβ_42_-induced cellular and membrane damages as shown by preserved normal cellular morphology (Fig. 2B) as well as reduced LDH release (Fig. 4C). These findings suggested the potential of compounds in stabilizing the cell membranes, preventing membrane leakage, and preserving cellular integrity. These effects could contribute to the maintenance of cellular homeostasis and protection against damages triggered by Aβ_42_. Furthermore, the compounds effectively protected against Aβ_42_-mediated mitochondrial dysfunction as demonstrated by preserved MMP, a key indicator of mitochondrial health and function (Fig. 4B). This protective property suggested that the compounds may prevent energetic deficits and maintain crucial cellular processes required for neuronal survival. The Aβ_42_ is well-known to induce oxidative damage in neurodegeneration. The selected compounds demonstrated abilities to counteract the Aβ_42_-induced oxidative stress as shown by their potent antioxidant capacities and abilities to diminish intracellular ROS levels (Fig. 4A). Additionally, these compounds not only directly scavenge the ROS, but also significantly enhance cellular antioxidant defense mechanisms by increasing catalase-like activity (Fig. 3B). This dual antioxidant action suggested their promising potentials as disease-modifying neuroprotective agents. In view of chemical structure and activity relationship (SAR) of the compounds, notable differences between active and inactive compounds were observed. The critical influence of R substituents (type and position) on the phenyl ring is highlighted. As observed for compounds **4** and **10**, and compounds **1** and **12**, which have the same electron withdrawing groups but different positions of NO_2_ and COCH_3_, respectively. Only compounds **10** and **12** demonstrated neuroprotective effects. This could be due to such property of functional groups at *meta*-position may be required for appropriate interaction with the site of neuroprotective action. These observations emphasize that types and substitution patterns of R substituents can significantly impact biological activity, guiding further structural optimization to enhance neuroprotective properties. However, further studies on SAR are required to gain deeper insights into the precise influence of these modifications. Moreover, this study was performed in SH-SY5Y cells. Even though they exhibit various neuronal features, they do not fully replicate the complexity of in vivo neuronal environments, such as the influence of glial cells and the extracellular matrix. Hence, the response of SH-SY5Y cells to Aβ may differ from primary neurons and potentially affect the generalizability of our findings.

In-depth neuroprotective mechanisms of these compounds were demonstrated by their modulating effects on BACE1 and SIRT1 activities. BACE1 enzyme cleaves the amyloid precursor protein and plays an essential role in the production of pathogenic Aβ [2]. BACE1 activity is normally found to increase in AD conditions, however, pretreatment with the selected compounds (**10**, **12**, **16**, and **18**) showed an effective decrease of BACE1 activity in the Aβ_42_-induced cells (Fig. 5). SIRT1 is well-known to play roles in neuroprotection *via* modulating several key neurodegeneration-related pathways such as decreasing the generation and aggregation of misfolded proteins (Aβ) and inhibiting the transcription of the BACE1 gene, resulting in a decline in BACE1 activity [77]. SIRT1 combats oxidative stress, neuroinflammation, and excitotoxicity through upregulating antioxidant proteins including FOXO3a, SOD2, and CAT [78, 79]. SIRT1 also promotes cellular health by activating autophagy, a process crucial for clearing damaged proteins and organelles, *via* modulating involved pathways (i.e., AMPK, HIF-1α, and PGC1α) [80, 81]. Furthermore, SIRT1 plays multifaceted roles in neuronal health (i.e., cellular metabolism, energy homeostasis, and cell membrane repair). Accordingly, activation of SIRT1 is actively pursued as a promising therapeutic strategy for managing neurodegenerative diseases [82]. Herein, it was demonstrated that cellular SIRT1 activity was decreased upon induction by Aβ_42_. Particularly, the selected compounds (**10**, **12**, **16**, and **18**) promisingly preserved SIRT1 activity with comparable potency to that of the RSV, the known SIRT1 activator (Fig. 6). This was supported by a molecular docking study which indicated that these compounds could suitably bind within the activator-binding site of the SIRT1 target in a similar manner with RSV as demonstrated by shared key interacting amino acid residues (i.e., ILE223 and ASN226). The bindings of these compounds were dominated by pi-interactions formed between the NQ core or substituted phenyl ring of the compounds and SIRT1. Additionally, these compounds showed superior binding affinity towards the SIRT1 as indicated by their lower binding free energy values (compounds from − 10.47 to − 9.34 kcal/mol and RSV − 7.51 kcal/mol) (Fig. 7A-D).

In recent years, AD has been recognized as the complexity of pathological processes including Aβ accumulation, tau hyperphosphorylation, neuroinflammation, and oxidative stress. Traditional drug discovery approaches have often focused on single-target therapies, which may not adequately address the multifaceted nature of the AD. With this context, multi-target drugs offer a promising strategy by simultaneously modulating several biological pathways, potentially leading to more effective therapeutic outcomes [83, 84]. This multi-target approach may enhance the efficacy of the ANQ derivatives in mitigating the progression of AD by addressing the disease from multiple angles. Therefore, in silico analysis was used to predict various potential targets associated with our investigated compounds, aiming to identify broader neuroprotective mechanisms, which were highlighted in a list of the top 20 potential AD-associated target proteins (Table 2). These targets play roles in neuroinflammation, neuronal cell death, neuronal functions, and the aggregation of misfolded proteins. Our network analysis unveiled a significant interconnectedness of the proteins within the network (Fig. 8B), which suggested that targeting these central hubs could have a cascading effect influencing multiple AD-related pathways. Accordingly, investigated compounds may be able to modulate the Aβ-induced neuroinflammatory response by targeting Src family kinases [50, 51]. Moreover, TNF is noteworthy for further investigation regarding the role of neuroinflammation in neurodegeneration [69]. Similarly, the JAK/STAT pathway plays a role in chronic neuroinflammation [85, 86]. This suggested that our compounds could be further developed for modulating pro-inflammatory cascades to combat neuroinflammation. Targeting estrogen signaling pathways has shown promise in AD research, particularly for female patients. ESR1 could be a promising target for personalized therapeutics [54]. Several targets relating the neuronal death were identified. Caspase 3 is a central player in neuronal apoptosis [71, 72]. Several identified proteins also intimately link to the MAPK pathway, which is implicated in neuronal cell death and AD progression [87, 88]. Lastly, the identified proteins influence the FOXO pathway (i.e., PI3K, JNK, MAPK, CREBBP, STAT, and EGFR), which suggests the protective possibilities against the devastating effects of this hallmark protein [86]. Interestingly, both MAPK and FOXO pathways are known to be regulated by SIRT1 activity [22, 89, 90]. This finding together with our in vitro results potentially suggested a unifying mechanism underlying the neuroprotective effects of the studied ANQs through the downstream targets of SIRT1. Taken together, the findings highlighted that the ANQs are promising compounds to be further developed as multifunctional neuroprotective agents to combat the multifaceted pathogenesis of AD. However, future studies regarding experimental target validation and functional studies to elucidate their impacts on relevant AD-related cellular processes are highly recommended.

Preferable pharmacokinetics and toxicity profiles are essential for successful drug development. In silico predictions demonstrated that all four compounds (**10**, **12**, **16**, and **18**) are drug-like compounds with favorable pharmacokinetics for potential oral administration (Table 1). Their moderate CNS and BBB permeabilities indicated their access to the target site in the CNS. However, all compounds are CYP3A4 substrates and some of them are inhibitors of other CYPs. This raised the concerning issue regarding their drug-drug or food-drug interaction potentials and further investigations are recommended. Although these compounds were predicted as moderately hazardous, it is suggested that in vitro and in vivo studies on hepatotoxicity, carcinogenicity, and other toxic issues should be further investigated.

## Conclusion

A series of 18 ANQ derivatives were comprehensively investigated for their neuroprotective potentials against AD in the Aβ_42_-induced SH-SY5Y cells. Among all tested compounds, four compounds (**10**, **12**, **16**, and **18**) at 1 µM were selected for further experiments due to their promising protective effects comparable to that of the RSV (84.61%). The structural features of these compounds are NQs with 2-aminophenyl containing R substituents (3-NO_2_, 3-COCH_3_, and 4-CH_3_)-3-chloro groups as noted for compounds **10**, **12**, **16**, and *R* = 4-OCH_3_ for compound **18**. The mechanisms underlying this protection were revealed *via* their abilities against intracellular ROS production and cellular membrane damage as well as maintaining mitochondrial function. Furthermore, the compounds effectively modulated BACE1 and SIRT1 activities, which highlighted the potential for the reduction of Aβ_42_ accumulation and other key harmful events. This was supported by the molecular docking study, which showed that these compounds (**10**, **12**, **16**, and **18**) are potential SIRT1 activators. The NQ core and the terminal substituted phenyl ring presented in the molecules were noted to be essential key structural features required for interactions with key interacting amino acid residues (i.e., ILE223 and ASN226). Target prediction indicated a top-ranked list of possible AD-related targets of these compounds, which demonstrated their potentials for combating the complexing of the disease in multiple facets (i.e., neuroinflammation, neuronal cell death, and misfolded proteins) under the SIRT1 regulation. Additionally, predictions of pharmacokinetics and toxicity profiles suggested that these compounds are drug-like molecules with CNS-penetrating ability as well as good intestinal absorption. Taken together, these ANQ compounds are promising candidates for further development as oral disease-modifying agents for the management of AD.

## Data Availability

The data and materials that support the findings of this study are available from the corresponding author upon reasonable request.
